# Calcineurin Inhibitors and Uric Acid Control in Solid Organ Transplantation: A Systematic Review

**DOI:** 10.3390/medsci14020191

**Published:** 2026-04-10

**Authors:** Francesca K. Martino, Marco Bogo, Ludovica Brunetta, Francesca Fioretti, Leda Cattarin, Lucia F Stefanelli, Federico Nalesso

**Affiliations:** Nephrology, Dialysis and Transplantation Unit, Department of Medicine (DIMED), University of Padova, 35128 Padua, Italy; marco.bogo@studenti.unipd.it (M.B.); ludovica.brunetta@studenti.unipd.it (L.B.); francesca.fioretti@studenti.unipd.it (F.F.); leda.cattarin@aopd.veneto.it (L.C.); luciafederica.stefanelli@unipd.it (L.F.S.); federico.nalesso@unipd.it (F.N.)

**Keywords:** uric acid, calcineurin inhibitors, cyclosporin, tacrolimus

## Abstract

**Background/Objectives**: Asymptomatic hyperuricemia has been associated with increased cardiovascular risk; it is related to factors such as diet, genetic predisposition, and drug-related side effects. Impairment of uric acid control has been associated with the calcineurin inhibitors cyclosporin and tacrolimus, although available studies did not reach the same conclusions. Their widespread use in solid organ transplantation potentially exposes this population to higher cardiovascular risk. This systematic review aimed to assess their role in hyperuricemia risk compared with other immunosuppressive treatments and to clarify potential differences between cyclosporin and tacrolimus. **Methods**: The search was conducted in MEDLINE and Embase, limited to adult subjects, using the following terms: ((cyclosporin) OR (cyclosporine) OR (tacrolimus) OR (calcineurin inhibitor)) AND ((uric acid) OR (urate) OR (hyperuricemia)) AND ((transplant) OR (transplantation)). We assessed the quality of the studies according to the Critical Appraisal Skills Programme checklist. **Results**: After screening 639 manuscripts, we selected 36 studies that were relevant to our focus: 28 evaluated kidney transplant patients, while only eight focused on other solid organ transplants. Specifically, 20 studies compared calcineurin inhibitors with other immunosuppressants, while 15 assessed the impact of cyclosporin versus tacrolimus, and one study contributed to both scenarios. The prevalence of hyperuricemia ranged from 30 to 80% among patients receiving calcineurin inhibitors, with a slightly higher prevalence with cyclosporin than with tacrolimus (51–61% vs. 36–42%, respectively). The overall quality of the included studies was generally rated as low to moderate, with only ten studies focusing on uric acid control. **Conclusions**: Given the heterogeneity and overall quality of the available studies, no definitive conclusions can be drawn. In particular, the comparative effect of cyclosporin and tacrolimus remains uncertain because of conflicting findings across studies. Although calcineurin inhibitors may adversely affect uric acid control in transplant recipients, this association may be influenced by several confounding factors.

## 1. Introduction

Asymptomatic hyperuricemia is defined by uric acid (UA) levels above 6.8 mg/dL (405 µmol/L) in the absence of inflammatory manifestations related to urate crystal deposition [1]. Conversely, gout is an inflammatory disease characterized by the deposition of monosodium urate crystals in joints and other tissues and by typical painful flares in the distal extremities [1,2,3]. The prevalence of asymptomatic hyperuricemia and gout has remained stable over the last few decades in the general population, estimated at around 20% and 4%, respectively, according to NHANES data [4]. These conditions are more common in men and older adults, with a prevalence of 11–13% among individuals aged 80 years and older [5,6,7]. High levels of UA often precede clinical manifestations of gout [8]. However, they are also linked to a wide range of pathological disorders, such as cardiovascular disease [9,10,11,12], metabolic syndrome [13,14,15], faster progression of chronic kidney disease (CKD) [16,17,18,19,20], and the development of preeclampsia during pregnancy [21,22,23]. Although the exact role of UA in these diseases’ development is not yet fully understood, increasing evidence suggests an association between UA and various pathological processes, including oxidative stress [24,25], endothelial dysfunction [26,27], inflammation [1], and impaired placental development [28].

Any condition that alters UA levels, including disorders of urate excretion or production, increases the risk of asymptomatic hyperuricemia and consequently of cardiovascular disease, metabolic syndrome, and kidney disease. Endogenous purine metabolism is the primary driver of hyperuricemia, while diet contributes only modestly to hyperuricemia [29,30]. In high-risk subjects, dietary interventions should include moderation of protein intake and avoidance of excessive intake of specific carbohydrates and fats that may increase serum urate levels [31,32]. In addition to diet, other modifiable factors can adversely affect UA control, including drug effects on UA renal excretion [33]. The kidney is an essential regulator of circulating UA levels by reabsorbing approximately 90% of filtered urate, accounting for 60–70% of total body UA excretion. Specifically, the kidney handles UA excretion via two cotransporters, URAT1 and GLUT9, which operate in the proximal tubule at the apical and basolateral membranes, respectively. Several drugs interfere with URAT1 and GLUT9, thereby modulating UA control, including SGLT2 inhibitors, losartan, pyrazinamide, and probenecid [34,35,36,37]. Other drugs, such as thiazides and loop diuretics, interfere with OAT1, OAT3, and OAT4, anion exchangers in the tubular cells, thereby increasing UA reabsorption [38]. Cyclosporin and tacrolimus seem to increase proximal tubular reabsorption of UA [39,40], especially in the setting of diuretic-induced volume depletion. Furthermore, they reduce glomerular filtration rate through afferent arteriolar vasoconstriction.

The incidence of hyperuricemia and gout has increased significantly among transplant recipients [41,42,43], possibly related, at least in part, to the widespread use of calcineurin inhibitors (CNIs). Currently, the available evidence provides an unclear picture of the impact of the CNI class on UA levels compared with other immunosuppressants, as well as of the cyclosporin- and tacrolimus-specific profiles on UA metabolism. In solid organ recipients, the UA abnormalities related to the use of CNIs could increase the risk of gout, hypertension, cardiovascular disease, metabolic syndrome, and CKD, and even of pre-eclampsia. The present systematic review aimed to describe the impact of the CNI class and the specific differences between cyclosporin and tacrolimus on UA levels, as a preliminary step toward optimizing UA control in solid organ recipients and reducing the potential contribution of immunosuppressive therapy to cardiovascular, metabolic, renal, and pregnancy-related complications.

## 2. Materials and Methods

This review was conducted in accordance with PRISMA guidelines [44], and a PRISMA 2020 checklist is reported in Appendix, Table A1. The protocol was registered on the Open Science Framework (OSF) website at https://osf.io/tukw3 (accessed on 15 December 2025).

Studies were included if they met the following PICO (population, intervention, comparison, outcome) criteria.

The following eligibility criteria were applied:

- Participants: Adult patients (≥19 years) who received cyclosporin or tacrolimus as immunosuppressive therapy for a solid organ transplant.
- Outcomes: UA levels or hyperuricemia incidence or prevalence in specific immunosuppressive regimens.
- Study Designs: Observational studies (prospective cohort studies, retrospective studies, and cross-sectional studies) and randomized controlled trials (RCTs).
- Report Characteristics: No language restrictions were imposed, and translations were attempted for non-English published articles. No time restriction was applied.

For the comparative component of the review, comparative studies were defined as studies that reported either: (1) within-patient comparisons, where UA levels or hyperuricemia were measured before and after starting, stopping, or changing cyclosporin or tacrolimus in the same patients; and (2) between-group comparisons, where UA levels or hyperuricemia outcomes were compared between patients on CNI regimens and those not on CNI regimens, as well as between patients taking cyclosporin and those taking tacrolimus. These two types of comparisons were described separately because they used different methods and carried different risks of bias.

To avoid double-counting, potentially overlapping study populations were identified by comparing study settings, recruitment periods, participating centers, authorship, sample sizes, and participant characteristics. When multiple reports described the same or overlapping populations, the most informative study was selected based on the completeness of outcome reporting, sample size, and follow-up duration.

Electronic searches

The search strategy was developed by F.K.M. and edited by F.K.M. and F.N. A search was conducted in MEDLINE and Embase (inception to September 2025, with no language restrictions) to identify eligible reports. Reference lists of relevant studies were screened. Search terms included ((cyclosporin) OR (cyclosporine) OR (tacrolimus) OR (calcineurin inhibitor)) AND ((uric acid) OR (urate) OR (hyperuricemia)) AND ((transplant) OR (transplantation)). Filters: Adult: ≥19 years.

Study selection

Four independent reviewers (M.B, L.B., F.F., and F.K.M.) screened titles and abstracts and independently inspected the full texts of potentially eligible observational studies to determine eligibility. Duplicate records were determined using EndNote version 25 (Clarivate Analytics, Philadelphia, PA, USA) and manual screening.

Assessment of heterogeneity

Clinical heterogeneity was described by comparing participant characteristics, baseline kidney function, other immunosuppressive treatments, transplant vintage, and duration of follow-up. Given the substantial heterogeneity across the included studies, quantitative synthesis was not performed; findings were synthesized narratively with a table.

Quality and relevance assessment

The methodological quality of the included studies was evaluated using CASP checklists specific to each study design: cohort, randomized controlled trial, and cross-sectional [45]. These checklists systematically assess study strengths and weaknesses by answering Yes, No, or Cannot tell to questions about methodological rigor, validity, clarity of results, and relevance to the review question. Due to substantial heterogeneity among the included studies, the quality assessment findings were summarized descriptively rather than synthesized into an overall quantitative rating.

Furthermore, to better characterize each study’s relevance to uric acid (UA), we assigned each study a score from 0 to 100, where 0 indicated no relevance and 100 indicated maximal relevance to UA-related outcomes. The score was established based on:

- The aim of the study: with a score from 0 to 20, where 0 was for a study that incidentally described UA metabolism, 10 was for a study that evaluated UA as a secondary outcome, and 20 was for a study that evaluated UA as a primary outcome.
- Study design and sample size: with a score from 0 to 20, which considered the type of study and the sample size.
- Accuracy of the description of UA or hyperuricemia, with a score from 0 to 20
- Accuracy of the description of kidney function at baseline and during the follow-up, with a score from 0 to 20.
- Accuracy in the potential biases that could have affected UA levels, with a score from 0 to 20. We focused on uricosuric agents, diuretics, antihypertensive treatment, and other immunosuppressive therapies.

Three reviewers (M.B., L.B., and F.F.) independently reviewed the articles. Any disagreements among the reviewers were resolved through group discussion and analysis (M.B., L.B., F.F., and F.K.M.) to reach consensus. A table of all included studies was created to describe each study’s potential bias and the UA relevance score for the presented quality data.

Data collection

Two reviewers (F.K.M. and L.F.S.) extracted data about the pertinent results. Information on outcomes and study characteristics was collected.

Outcome measures

In the comparative studies, we identified differences in UA levels as the outcome measure, as defined in each study. Ultimately, we considered an adequate outcome to be the prevalence or incidence of hyperuricemia, or the odds ratio for hyperuricemia.

In cohort studies and RCTs, we examined changes in UA levels before and after initiation or discontinuation of cyclosporin or tacrolimus, with UA as the outcome measure. Additionally, in these study types, we considered the incidence, odds ratio, or hazard ratio for hyperuricemia as an adequate outcome measure. The definition of hyperuricemia was reported for each study, as specified in the original manuscript.

Certainty assessment

We used the GRADE (Grading of Recommendations Assessment, Development, and Evaluation) approach to assess the evidence and the strength of recommendations [46]. Because of substantial clinical heterogeneity and the absence of quantitative pooling, GRADE judgments were made for narrative syntheses for all the following features: study design, risk of bias, inconsistency, indirectness, imprecision, and publication bias.

Data synthesis

All included studies were listed in a table that described the year of publication, study type, and sample size. Furthermore, the studies were organized into two tables: one comparing UA outcomes between CNI and other immunosuppressive treatments, and another evaluating changes in UA between cyclosporin and tacrolimus. Within each table, studies were further grouped by comparison type. For each study, we provide a brief description of the design, sample size, study population characteristics, and results.

The quality assessment of the included studies was summarized in a table that describes the reviewers’ evaluation according to the CASP checklist and the relevance score of UA presentation.

Finally, an additional table summarized, for each study, population characteristics, baseline kidney function, concomitant immunosuppressive therapy, transplant vintage, follow-up duration, and the definition of hyperuricemia, to highlight heterogeneity across the included studies.

## 3. Results

**Figure 1:**
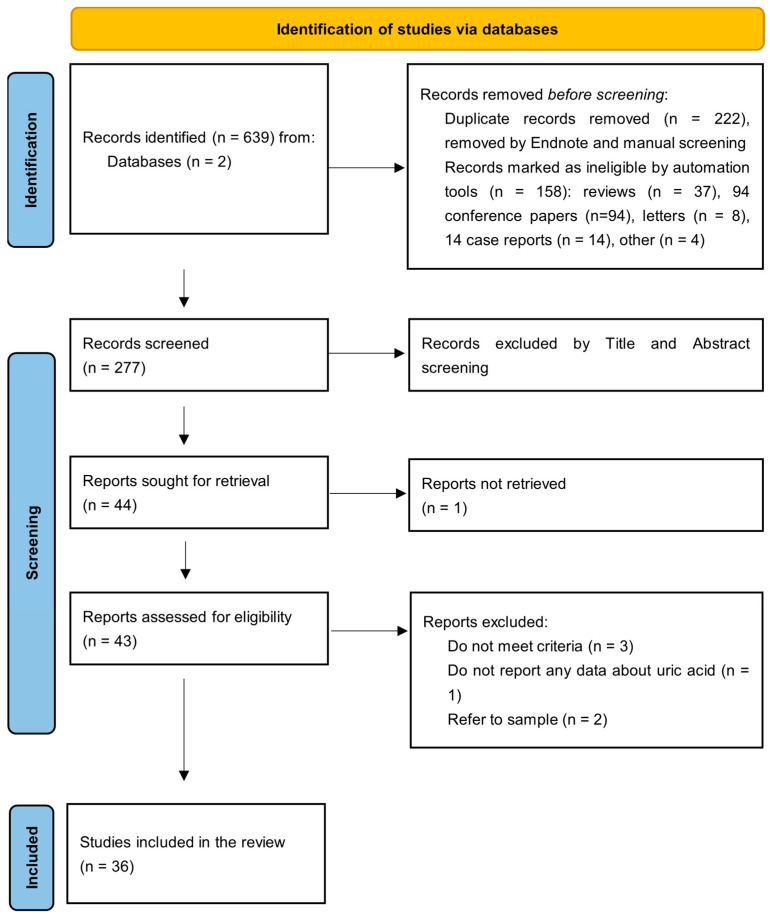
Screening procedure of manuscripts.

Initially, 639 manuscripts were identified; 158 were excluded due to the type of publication, 222 were excluded as duplicates, as determined using EndNote 2025 and manual screening, and 277 were analyzed based on title and abstract. A total of 43 full-text articles were identified in accordance with the screening criteria, of which 36 were relevant to our focus. Notably, two studies that referred to the same sample were counted only once. The specific procedure for the literature screening is presented in Figure 1.

The principal characteristics of all studies included according to the following criteria (“cyclosporin” OR “cyclosporine” OR “tacrolimus” OR “calcineurin inhibitor”) AND (“uric acid” OR “urate” OR “hyperuricemia”) AND (“transplant” OR “transplantation”) were summarized in Table 1.

### 3.1. Uric Acid Control in the Included Studies

Thirty-six studies evaluated the relationship between UA and CNI; of these, twenty-one compared UA with other immunosuppressive regimens, and sixteen examined the role of cyclosporin versus tacrolimus, totaling 9029 cases. Notably, Claes K et al. explored both comparisons [4].

#### 3.1.1. Uric Acid in Cyclosporin or Tacrolimus Treatment Versus Other Immunosuppressives

Twenty-one studies assessed the impact of cyclosporin or tacrolimus on UA levels between 1985 and 2022. These included six prospective, four retrospective, and three cross-sectional studies, along with eight RCTs, involving a total of 7767 cases. Two studies focused on liver transplant patients, two focused on heart transplant patients, and the rest examined UA effects in kidney transplant patients. Only eight studies used UA levels as a primary endpoint, while the others evaluated the effects of cyclosporin or tacrolimus on UA levels as a secondary outcome. Specifically, six studies investigated the prevalence of hyperuricemia in patients receiving CNI treatment; five studied UA levels following CNI dose adjustments; five compared CNI with other immunosuppressants regarding UA control; three examined the impact of switching from CNI to other immunosuppressants and vice versa; and two explored UA effects after CNI withdrawal. The follow-up duration ranged from less than 1 month to over 8 years.

**Figure 2:**
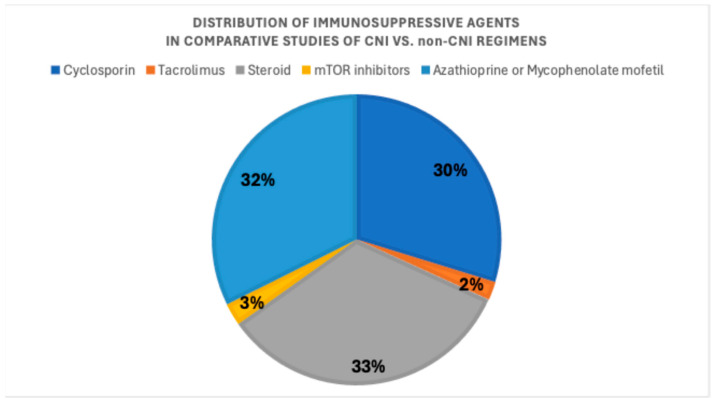
Prevalence of immunosuppressive agents across the studies that compared CNI versus non-CNI regimens.

Most studies involved patients receiving cyclosporin, antimetabolites, and steroids. Figure 2 shows the prevalence of immunosuppressive treatments across studies; two studies did not provide detailed information about their immunosuppression treatments.

**Figure 3:**
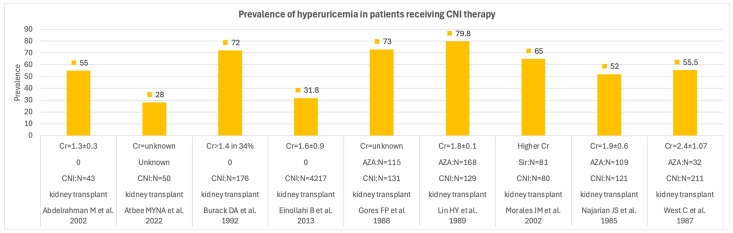
Prevalence of hyperuricemia during CNI therapy across solid organ transplant recipients [47,48,49,50,52,60,61,77,81]. Cr = creatinine (mg/dL), N = number of cases, AZA = azathioprine, Sir= sirolimus.

The prevalence of hyperuricemia, which had varying definitions across the studies, was quite common among solid organ transplant recipients who received cyclosporin or tacrolimus as immunosuppressants. Although there was a significant variation in the prevalence of hyperuricemia across the studies, it ranged from 30 to 80% in patients who received CNI. Figure 3 shows the prevalence across these studies.

**Figure 4:**
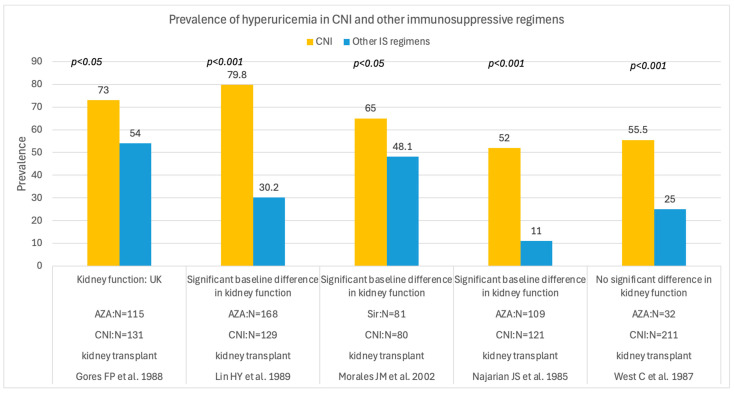
Prevalence of hyperuricemia in kidney transplant recipients receiving CNI and other immunosuppressive regimens [47,48,49,50,61]. CNI = calcineurin inhibitor regimens; IS = immunosuppressive regimens; N = number of patients; UK = unknown; AZA = azathioprine; Sir = sirolimus.

The prevalence of hyperuricemia was lower in patients receiving azathioprine or sirolimus than in those receiving CNI therapy, as shown in Figure 4.

**Figure 5:**
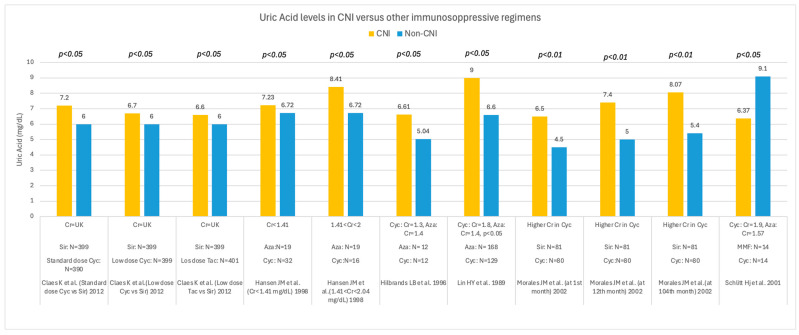
Uric acid levels in solid organ recipients receiving CNI versus other immunosuppressive regimens [50,55,56,59,61,76]. UA is reported in mg/dL; Cr = creatinine (mg/dL); CNI = calcineurin inhibitor; Cyc = cyclosporin; Tac = tacrolimus; Sir = sirolimus; AZA = azathioprine.

Some studies have shown a significant impact of cyclosporin levels on UA control, indicating that UA levels can be affected by cyclosporin dosing. The same conclusion was reached by studies on CNI withdrawal and dose reduction, which showed improved UA control. Figure 5 displays the results of studies comparing UA levels between CNI and non-CNI regimens in a non-paired series. Only two studies examined the switch from CNI to other immunosuppressive treatments, Bumbea et al. [67] and Chen et al. [72], with contrasting results. Bumbea et al. reported a significant reduction in UA after CNI withdrawal, which persisted throughout follow-up, whereas Chen et al. did not observe a difference in UA levels after CNI discontinuation.

In this context, we would emphasize that, among patients receiving CNI as immunosuppressive therapy, some studies have demonstrated a consistent link between kidney function and diuretic use with UA control, as well as a higher prevalence of hyperuricemia in males compared to females. Notably, none of these potential confounders were systematically examined in the studies reviewed. Table 2 summarizes the key characteristics of each study, including the population, follow-up, and main findings.

#### 3.1.2. Uric Acid in the Comparison Between Cyclosporin and Tacrolimus

**Figure 6:**
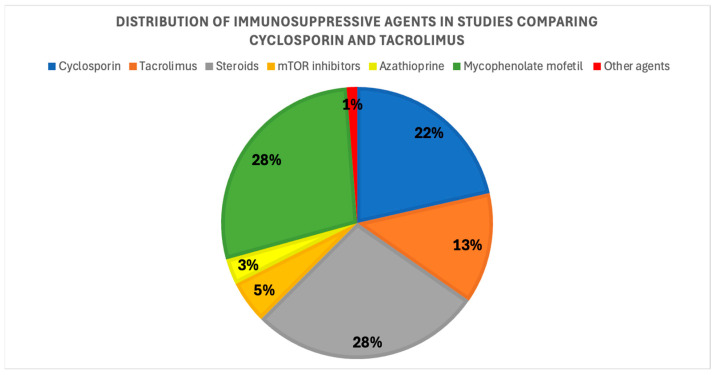
Prevalence of immunosuppressive agents across studies comparing cyclosporin and tacrolimus.

Sixteen studies analyzed the effects of cyclosporin and tacrolimus on UA levels from 1994 to 2020, including a total of 3168 patients. Among these, three were prospective cohort studies, seven were retrospective studies, two were cross-sectional studies, two were RCTs, and two had no clear classification. One study focused on heart transplant patients; two on liver transplant patients; one on both heart and liver transplant patients; and twelve on kidney transplant patients. Additionally, only three studies considered UA control during the switch from cyclosporin to tacrolimus as the primary outcome; the remaining studies regarded UA as a secondary outcome. The follow-up duration ranged from 2 weeks to over 7 years. The prevalence of immunosuppressive agent use is illustrated in Figure 6. In four studies, only cyclosporin and tacrolimus were reported, without details on other immunosuppressive treatments.

**Figure 7:**
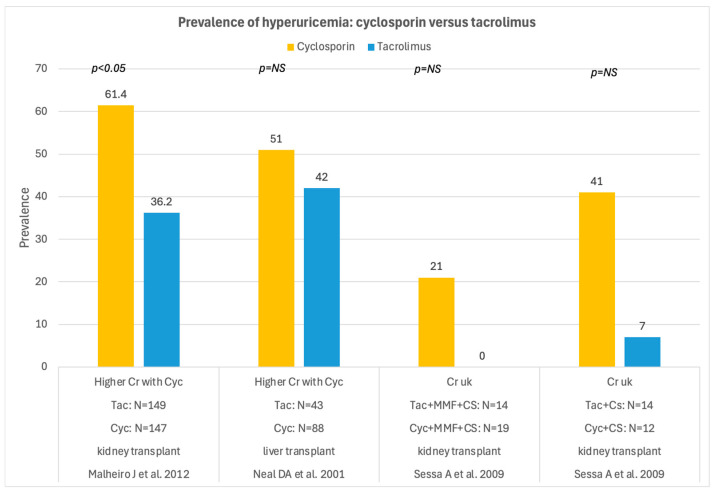
Prevalence of hyperuricemia in patients receiving cyclosporin versus tacrolimus after solid organ transplantation [43,58,74]. N = number of cases, Cyc = cyclosporin, Tac = tacrolimus, NS = not significant.

The prevalence of hyperuricemia did not differ significantly between cyclosporin and tacrolimus, although not all studies reached the same conclusion, as shown in Figure 7.

**Figure 8:**
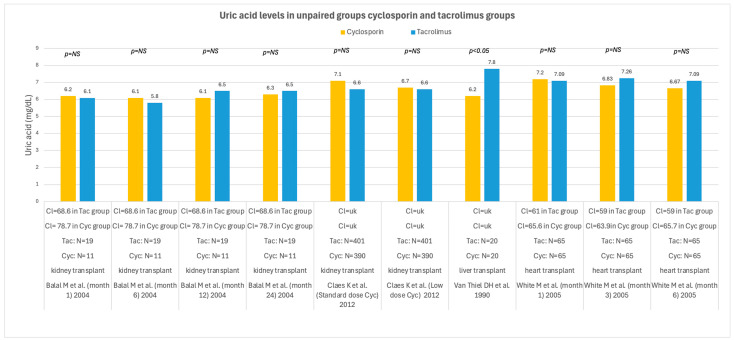
Uric acid levels in solid organ transplant recipients receiving cyclosporin or tacrolimus [51,64,71,76]. UA levels are reported in mg/dL; Cl = creatinine clearance (mL/min); N = number of patients; Cyc = cyclosporin; Tac = tacrolimus; NS = not significant.

**Figure 9:**
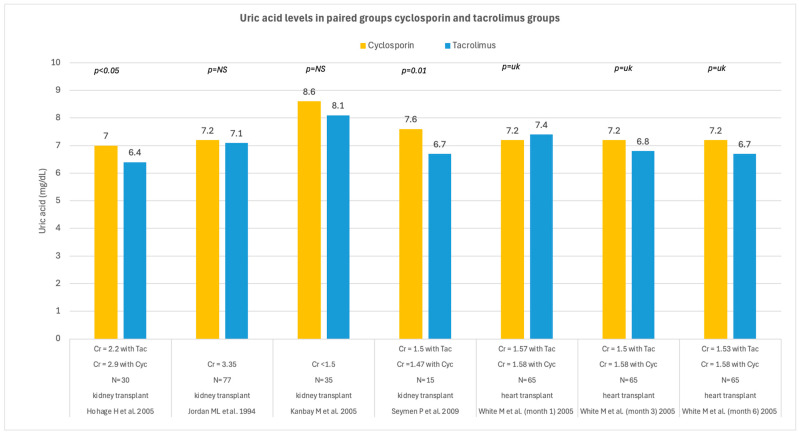
Uric acid levels in paired solid organ transplant recipients switched from cyclosporin to tacrolimus [53,68,69,71,75]. UA levels are reported in mg/dL; Cr = serum creatinine (mg/dL); N = number of patients; Cyc = cyclosporin; Tac = tacrolimus; NS = not significant.

In recipients of solid organs, cyclosporin and tacrolimus had different effects on UA levels during follow-up. Seven studies reported an association between cyclosporin use and higher UA levels compared with tacrolimus, whereas only two studies linked tacrolimus to worse UA control; seven additional studies found no significant difference between the two drugs. Figure 8 shows all studies comparing UA levels in solid organ recipients treated with cyclosporin versus tacrolimus, while Figure 9 depicts all studies evaluating the switch from cyclosporin to tacrolimus. The conversion from cyclosporin to tacrolimus generally showed a trend toward reduced or stable UA levels during follow-up, although two studies did not find a difference, and one study indicated a detrimental effect on UA after conversion.

Finally, in comparative studies between cyclosporin and tacrolimus, the UA control seems to worsen over time. All details about the studies are reported in Table 3.

### 3.2. Quality, Relevance and Heterogeneity Assessment

Twenty-six studies assessed UA control as a secondary outcome, while only ten examined it as the primary outcome. Their sample sizes varied from small to large, and their overall quality was generally moderate. Among the included studies, 13 had sample sizes less than 50; five had sample sizes between 50 and 100; seven had sample sizes between 100 and 1000; and two had sample sizes over 1000. The type of study, study design, sample size, and UA relevance score are summarized in Table 4.

The quality and relevance assessment of the included studies are summarized in Table 5. Only six studies had a high-quality profile according to CASP quality assessment, while the median relevance score for evaluating the impact of CNI on UA metabolism was 53.5 (IQR 42.5–62). Furthermore, in Table 6, we reported a systematic evaluation of confounding variables, such as kidney function, diuretics, other immunosuppressive therapy, other potential medications affecting UA control, and diet.

Most studies focused on kidney transplant patients; only eight examined the effects of cyclosporin or tacrolimus for liver or heart transplants. The authors did not always provide detailed information about immunosuppressive therapy, and when they did, the details varied between studies. The duration of the observation period ranged from less than one month to over ten years; patients were enrolled immediately after the transplant or several years later, and baseline kidney function, when reported, varied from normal to severely impaired. The assessment of heterogeneity across all studies is shown in Table 7.

Overall, according to the GRADE evaluation, we found very low certainty of evidence for both hyperuricemia and serum UA outcomes in both comparisons of immunosuppression regimens (CNI versus non-CNI and cyclosporin versus tacrolimus), as described in Table 8.

## 4. Discussion

To our knowledge, this is one of the first systematic reviews to explore the relationship between CNI treatment in solid-organ transplant recipients and UA control. It included 36 studies with 9029 participants. A total of 21 studies, involving 7767 transplant patients, examined the effects of cyclosporin or tacrolimus on UA, and 16 studies, totaling 3168 cases, compared the effects of cyclosporin and tacrolimus. Notably, Claes K et al. assessed both comparisons [76]. The majority of the included studies evaluated kidney transplant patients; eight evaluated heart or liver transplant recipients. The treatment with CNI seemed to worsen UA control, with a higher prevalence and incidence of hyperuricemia during the follow-up period. Furthermore, cyclosporin appears to be more strongly associated with hyperuricemia than tacrolimus, although the findings were inconsistent. Unfortunately, the poor quality of the studies, the high number of retrospective studies, the variability in follow-up duration and posttransplant observation periods, the incomplete assessment of kidney function, and the lack of evaluation of additional potential confounding factors all diminish the reliability of the conclusions.

### 4.1. CNI and Hyperuricemia

Hyperuricemia is quite prevalent in solid organ transplant recipients who were treated with CNI, and across the studies it ranged between 30% [77,81] and 80% [50], according to the type of patient and the definition. This association may have a physiological basis, reflecting the effects of CNIs on glomerular filtration and tubular urate transport [50,83,84,85,86], which suggests that hyperuricemia in solid organ transplant recipients may be related to CNI-induced kidney dysfunction [87,88]. This observation is also supported by comparison studies between CNI and non-CNI regimens (sirolimus [61] and azathioprine [47,48,49,50]), in which cyclosporin and tacrolimus were associated with a significantly higher prevalence of hyperuricemia, with concomitantly worse kidney function. Unfortunately, in some studies, kidney function was poorly described, limiting the reliability of the results. Furthermore, we observed wide variability in prevalence, which could be related to differences in the cut-off used to define hyperuricemia and to heterogeneity in kidney function, other pharmacological treatments, and transplant periods.

Data on the prevalence of hyperuricemia generally focus on kidney transplant patients; only one study examined its prevalence in heart transplant recipients [52], showing a high and comparable rate. Although in theory other solid organ transplants might have similar prevalence due to the physiological mechanisms of hyperuricemia in CNI treatment [41,89,90,91], the significant differences in the baseline conditions unique to each type of solid organ transplant [65,92,93,94], the underlying disease causing organ failure [21,95,96,97,98,99], the potential variation in the desired levels of CNI [100,101,102,103], and the combined effects of other medications [34,36,104,105,106] limit the general applicability of the findings from kidney transplant data.

### 4.2. CNI and UA Control

Cyclosporin or tacrolimus generally showed a consistent impact on UA control compared with other immunosuppressive regimens [50,54,56,61,76]. Switching from CNI to non-CNI regimens [55,56,57], or vice versa, and reducing [66,73,74,75,76,77,78] or discontinuing CNI [59,62] were associated with improved UA levels during follow-up. All these observations highlight not only the impact of CNI use as an immunosuppressive treatment, but also the role of CNI level in UA control. The lack of a clear message across the studies could be related to the different CNI levels, given that most studies fail to describe CNI levels. However, the relationship between CNI dose and the acute and chronic nephrotoxicity remains controversial [107].

Across these studies, the effect of CNI reduction or discontinuation on UA metabolism was generally accompanied by improvement in kidney function [47,50,59,61,66,67,73]. Remarkably, in kidney transplant recipients with normal or slightly reduced kidney function, stopping or lowering CNI significantly improved UA levels [55,56,59,60,62,66,67,70,73], whereas in those with moderate to severe kidney impairment, the effect of CNI dosing on UA appears less pronounced [72]. Likely, moderate-to-severe kidney function could be a stronger predictor of UA imbalance and could nullify the potential effect of CNI on UA metabolism. On the basis of these observations, kidney function evaluation may be recommended for all solid organ transplant recipients, to better understand the possible effects of CNI on UA metabolism, with a frequency aligned with kidney function assessment after the transplant and with the rate of progression of kidney impairment.

If kidney function and its change over time are possible confounding factors in analyzing the effect of CNI on UA metabolism, other factors could significantly impact UA control, such as the use of diuretics, the presence of uricosuric drugs, other medications that directly interfere with UA metabolism or CNI metabolism, and finally, patients’ dietary habits. We would highlight the lack of systematic assessment of other major confounding factors; only a few studies considered their effects on UA control, which limits the robustness of the evidence.

### 4.3. Cyclosporin Versus Tacrolimus in UA Control

Results regarding UA control in studies comparing cyclosporin and tacrolimus in solid-organ transplant patients were inconsistent. Seven studies showed higher UA levels with cyclosporin [44,63,68,69,71,75,76], and seven did not show any significant difference between the two regimens [51,53,57,64,74,79,80]. Despite inconsistent evidence across comparative studies, a trend toward improved UA has been observed in studies on cyclosporin discontinuation [53,68,69,71], indicating reductions or stabilization of UA levels over time and a potential benefit of stopping cyclosporin in favor of tacrolimus. Given the controversial results across studies and the limitations in assessing UA control, it remains unclear whether tacrolimus has a more favorable effect on UA handling, despite the differential effects of these agents on glomerular and tubular function as described in previous studies [107,108,109]. Cyclosporin may have a greater impact on the proximal tubular segment [110,111], potentially increasing UA even in the early stages of CKD, whereas tacrolimus may negatively affect UA control by impairing glomerular filtration [108,112], However, this interpretation is only partially supported by the available clinical evidence.

### 4.4. Clinical Implications

Asymptomatic hyperuricemia is common among solid organ recipients. It serves as a risk factor for kidney impairment and progression, as well as for developing cardiovascular diseases such as hypertension, coronary heart disease, heart failure, and stroke [10,113], as well as metabolic disorders like diabetes and obesity [114]. Specifically, in a solid organ transplant recipient, existing conditions related to organ failure could exacerbate the development of such complications and create a harmful synergy with UA abnormalities. In patients receiving CNI treatment, understanding the evidence on the relationship between CNI and UA control is important for managing cardiovascular, kidney, and metabolic risks. Our results seem to indicate a relationship between CNI and hyperuricemia, as well as between CNI levels and UA levels. This may justify closer monitoring and individualized dose adjustment in selected high-risk patients. While current data do not clearly establish the roles of cyclosporin and tacrolimus in UA control, they suggest a possible, though not certain, benefit of tacrolimus over cyclosporin. Therefore, switching from cyclosporin to improve UA control is not justified based on the current evidence. Furthermore, given the relationship between kidney function and UA levels, monitoring UA levels over time should be considered, especially in patients with advanced CKD, where discontinuing CNI or switching from cyclosporin to tacrolimus may only marginally improve UA control. In any case, changing immunosuppressive strategies to manage hyperuricemia should consider the risk of rejection and potential worsening of metabolic conditions.

### 4.5. Limitations and Quality Assessment

This systematic review aimed to assess the evidence on the role of CNI treatment in UA control in solid organ recipients; unfortunately, the findings do not allow for definitive conclusions. This result is mainly due to the low methodological quality and high heterogeneity of the studies. As reported in Table 6 and Table 7, the quality analysis of the studies showed that only six were of good quality according to the CASP questionnaire, a high prevalence of retrospective studies, and high heterogeneity across studies in terms of time after organ transplant, sample size, and other immunosuppressive regimens. Furthermore, the quality of the data on UA metabolism was assessed by a non-validated tool. Still, during the evaluation of the studies, we found a discrepancy between the quality assessment of the studies and the relevance of the data referring to UA metabolism. The median UA relevance score was 50 (IQR 42.5–62), indicating an overall suboptimal assessment of UA-related outcomes, particularly because major confounding factors were often not adequately evaluated. This weakness could be related to the fact that UA was the primary outcome in only 10 studies.

Given the high heterogeneity in study characteristics, differences in study period, and differences in the definition of hyperuricemia, we decided not to proceed with a meta-analysis [115,116]. In this context, RCTs with a detailed evaluation of confounding factors are needed to assess the true impact of CNI on UA metabolism in solid-organ recipients. Specifically, given the importance of kidney function in determining UA control and the impact of CNI treatment on kidney function and UA metabolism, future studies should also consider using markers to detect tubular damage.

## 5. Conclusions

In solid organ transplant recipients, the high heterogeneity of studies regarding the definition of hyperuricemia, kidney function during follow-up, and study quality precludes definitive conclusions. CNI treatment appears to be associated with a high prevalence of hyperuricemia across studies. Furthermore, it could affect UA control depending on the drug dose and the patients’ kidney function. Yet, it remains unclear whether cyclosporin and tacrolimus have different effects on UA control and to what extent kidney function influences UA metabolism during CNI treatment. However, monitoring UA levels during post-transplant follow-up should be considered for all solid organ recipients in order to optimize immunosuppressive treatment and reduce the risk of hyperuricemia.

## Figures and Tables

**Table 1:** Principal characteristics of studies.

Authors	Year	Study Design	Control Type	Study Timing	Sample Size (*n*)
**Najarian JS et al.** [47]	1985	RCT	Parallel control group	Prospective	230
**West C et al.** [48]	1987	Cohort	Controlled	Retrospective	243
**Gores FP et al.** [49]	1988	RCT	Parallel control group	Prospective	246
**Lin HY et al.** [50]	1989	Cross-sectional	Controlled	Cross-sectional	297
**Van Thiel DH et al.** [51]	1990	Cohort	Historical control	Prospective	40
**Burack DA et al.** [52]	1992	Cross-sectional	No control group	Cross-sectional	196
**Jordan ML et al.** [53]	1994	Cohort	Controlled	Retrospective	77
**Islam IS et al.** [54]	1995	Cohort	Controlled	Retrospective	26
**Hilbrands LB et al.** [55]	1996	RCT	Parallel control group	Prospective	21
**Hansen JM et al.** [56]	1998	Cross-sectional	Controlled	Cross-sectional	111
**Boots JMM et al.** [57]	2001	Cohort	Controlled	Not clearly reported	128
**Neal DA et al.** [58]	2001	Cohort	Controlled	Retrospective	134
**Schlitt HJ et al.** [59]	2001	RCT	Parallel control group	Prospective	28
**Abdelrahman M et al.** [60]	2002	Cohort	No control group	Retrospective	45
**Morales JM et al.** [61]	2002	RCT	Parallel control group	Prospective	161
**Pascual M et al.** [62]	2003	RCT	Parallel control group	Prospective	64
**Urbizu JM et al.** [63]	2003	Cohort	Controlled	Retrospective	55
**Balal M et al.** [64]	2004	Cohort	Controlled	Prospective	30
**Shibolet O et al.** [65]	2004	Cohort	Controlled	Retrospective	122
**Wong V et al.** [66]	2004	RCT	Parallel control group	Prospective	31
**Bumbea V et al.** [67]	2005	Cohort	Controlled	Prospective	43
**Hohage H et al.** [68]	2005	Cohort	Controlled	Retrospective	30
**Kanbay M et al.** [69]	2005	Cohort	Controlled	Retrospective	155
**Paydas S et al.** [70]	2005	Cohort	Controlled	Prospective	54
**White M et al.** [71]	2005	RCT	Parallel control group	Prospective	129
**Chen J et al.** [72]	2008	Cohort	Controlled	Not clearly reported	16
**Pons JA et al.** [73]	2009	Cohort	Controlled	Prospective	20
**Sessa A et al.** [74]	2009	Cross-sectional	Controlled	Cross-sectional	103
**Seymen P et al.** [75]	2009	Cohort	Controlled	Prospective	15
Claes K et al. * [76]	2012	RCT	Parallel control group	Prospective	1645
**Malheiro J et al.** [43]	2012	Cross-sectional	Controlled	Cross-sectional	302
**Einollahi B et al.** [77]	2013	Cohort	No control group	Retrospective	4217
**Faulhaber M et al.** [78]	2013	Cohort	Controlled	Prospective	23
**Harada S et al.** [79]	2017	Cohort	Controlled	Not clearly reported	37
**Azizzadeh L et al.** [80]	2020	Cohort	Controlled	Retrospective	166
**Atbee MYNA et al.** [81]	2022	Cohort	No control group	Prospective	50

* Sub-analysis of Symphony study.

**Table 2:** Description of studies that evaluated cyclosporin or tacrolimus in comparison with other immunosuppression treatments regarding UA control in patients after solid organ transplantation.

Study ID	Aim and Population	Follow-Up (Months)	Study Groups	Kidney Function	Results
**Within-patient comparisons**					
**Hilbrands 1996** [55]	Renal function in first month after KTx	1	A: Cyc -> Cyc (N = 9) B: Cyc -> Aza (N = 12)	Cr = A: 1.31 ± 0.28, B: 1.41 ± 0.46	UA = A: 6.56 ± 1.51 -> 7.06 ± 1.01, *p* = NS B: 6.56 ± 1.51 -> 5.04 ± 1.01, *p* = 0.002, A 7.06 ± 1.01, B 5.04 ± 1.01, *p* < 0.05
**Pascual 2003** [62]	Safety after 50% Cyc reduction in KTx	≥6	A: Cyc full dose -> 50% Cyc reduction + CS + MMF (N = 32) B: Cyc full dose -> Cyc full dose (levels 100–300 ng/mL) + CS + MMF (N = 32)	Cr < 2 mg/dL	UA = A: 6.9 ± 1.7 -> 6.3 ±1.5, *p* = 0.04 B: 6.8 ± 1.5 -> 6.9 ±1.6, *p* = NS
**Wong 2004** [66]	CV risk after 50% Cyc reduction in KTx	6	A: Cyc full dose -> Cyc full dose (N = 15) B: Cyc full dose -> 50% Cyc reduction (N = 16)	Cr = A: 1.33 ± 0.2, B: 1.53 ± 0.23, *p* = 0.01	UA = A: 6.7 ± 1.6 -> 7.2 ± 1.5, *p* = 0.018 B: 6.9 ± 1.7 -> 6.4 ± 1.6, *p* = 0.013
**Bumbea 2005** [67]	Efficacy and safety after conversion from CNI to Sir: in chronic allograft dysfunction KTx	24	A: CNI (Cyc, 65%; Tac 35%) -> B: Sir (N = 43)	Cl = 49.4 ± 14.9	UA = 1 month: A: 7.3 ± 2, B: 6.5 ± 1.8, 1 year: A: 7.3 ± 2, B: 6.4 ± 1.7 2 years: A: 7.3 ± 2, B: 6.7 ± 1.8, *p* = 0.004
**Paydas 2005 (^)** [70]	Effects of C0 vs. C2 Cyc monitoring in KTx	36	Ac: C0 -> Bc: C2 (N = 12)	Cl = A: 72.31 ± 23.1, B: 78.73 ± 22.42, *p* = 0.621	UA = 12 months: Ac: 8.9 ± 0.7 -> Bc: 6.9 ± 0.4, *p* = 0.015 36 months: Ac: 8.9 ± 0.7 -> Bc: 7.1 ± 0.5, *p* = 0.011
**Chen 2008** [72]	Effect of Conversion from CNI to Sir in KTx with chronic allograft nephropathy	12	A: CNI -> B: Sir (N = 16)	Median Cr = 3.2	UA = A: 7 ± 2.25 B: after 3 months: 6.8 ± 2.3, after 6 months: 6.3 ± 0.9, after 12 months: 6.5 ± 1.36, *p* = NS
**Faulhaber 2013** [78]	CS withdrawal and Cyc reduction in HTx	24	A: Cyc + CS -> B: Cyc reduction (level of 50–90 ng/mL) + MMF (N = 23)	Cr < 3.5 mg/dL	UA = A: 7.6 ± 1.7 -> B: 5.9 ± 1, *p* < 0.001
**Between-group comparisons**					
**Najarian 1985** [47]	Side effects of immunosuppressor in KTx	3–36	A: Cyc + CS (N = 121) B: Aza + CS + Anti-Ly (N = 109)	Cr = A:1.9 ± 06, B: 1.5 ± 0.4, *p* < 0.001	HU = A: 52%, B: 11%, *p* < 0.001
**West 1987** [48]	Prevalence of gout and HU in KTx	≥12	A: Cyc + CS (N = 211) B: Aza + CS (N = 32)	Cr = A: 2.4 ± 1.07, B: 2.5 ± 1.25, *p* = NS	HU = 55.5% of A vs. 25% of B, *p* < 0.01
**Lin 1989** [50]	Prevalence and mechanism of HU in KTx	Not applicable	A: Cyc (N = 129) B: Aza(N = 168)	Cr = A: 1.8 ± 0.1, B: 1.4 ± 0.1, *p* = 0.0001	UA = A: 9.0 ± 0.2 B: 6.6 ± 0.1, *p* = 0.001 HU: A: 79.8%, B: 30.2%, *p* < 0.001 Diuretics increase HU prevalence in both groups
**Burack 1992** [52]	Prevalence of HU and gout in HTx	5–29	A: Gout (N = 14) B: Probable gout (N = 7) C: No gout (N = 157)	Cr > 1.4 mg/dL in 34% of patients	HU = ♀: 81%, ♂: 72%, A: 100% were in Cyc, and diuretics B: 100% were in Cyc, and diuretics C: 99% were in Cyc, and 95% in diuretics
**Islam 1995** [54]	Long-term Cyc biochemical effects in KTx	≥48	A: Cyc + Aza + CS (N = 13) B: Aza + CS (N = 13)	Not reported	UA = A: 6.56 ± 1.88, B: 5.5 ± 1.48, *p* < 0.05
**Hilbrands 1996** [55]	Renal function in first month after KTx	1	A: Cyc (N = 9) B: Cyc -> Aza (N = 12)	Cr = A: 1.31 ± 0.28, B: 1.41 ± 0.46	UA = A 7.06 ± 1.01, B 5.04 ± 1.01, *p* < 0.05
**Gores 1988** [49]	HU after KTx	48	A: Cyc + CS (N = 131) B: Aza + CS + Anti-Ly (N = 115)	Cr < 2	HU = A: 96 (73%), B: 87 (54%), *p* < 0.05. Severe HU: A: 13 (10%), B: 0 (0%), *p* < 0.002.
**Hansen 1998** [56]	Effect of low-dose Cyc on tubular function in KTx	Not applicable	A: Cyc (levels < 125 umol/L) (N = 32) B: Cyc with (levels 125–180 umol/L) (N = 16) C: Aza (N = 19) D: Control (N = 34)	Cr < 2	UA = A: 7.23 ± 1.5, B: 8.41 ± 2, *p* < 0.05 A: 7.23 ± 1.5, D: 4.9 ± 0.5, *p* < 0.05 B: 8.41 ± 2, C: 6.7 ± 1.2, *p* < 0.05 B: 8.41 ± 2, D: 4.9 ± 0.5, *p* < 0.05 C: 6.7 ± 1.2, D: 4.9 ± 0.5, *p* < 0.05 Cl related to UA-Cl with rho = 0.79, *p* < 0.001
**Schlitt 2001** [59]	Effect of CNI withdrawal and MMF replacement in Stable LTx with CNI toxicity	6	A: CNI -> MMF (N = 14) B: CNI -> CNI (N = 14)	Cr = A: 1.57 ± 0.18, B: 1.9 ± 0.58	ΔUA = A: −1.34 (−0.56 to −2.15), B: −0.04 (−0.9 to 1), *p* < 0.05
**Abdelrahman 2002** [60]	Prevalence of HU and associated factors in KTx after at least 12 months	≥106	Cyc (N = 43), No Cyc (N = 2) A:HU (N = 25), B: Non-HU (N = 20)	Cr = 1.3 ± 0.3	HU = 55% UA = A: 9.6 ± 1.4 with Cyc = 212 ± 45 ng/mL, B: 6.5 ± 0.9 with Cyc = 203 ± 41 ng/mL *p* = NS UA vs. Cyc dose: rho 0.1 *p* = NS UA vs. time after KTx: rho 0.01 *p* = NS
**Morales 2002** [61]	Impact on GFR of Cyc vs. Sir in KTx	104	A: Cyc + Aza or MMF (N = 80) B: Sir + Aza or MMF (N = 81)	Cr significantly lower in B	UA = 1 month: A: 6.05 ±0.01, B: 4.54 ± 0.01, *p* < 0.01 12 months A: 7.4 ± 0.34, B: 5.04 ± 0.34, *p* < 0.01 104 months: A: 8.1 ± 0.34, B: 5.4 ± 0.34, *p* < 0.01 HU in the first 3 months: A 65%, B 48.1%, *p* < 0.05 after 3 months: A 52.4%, B 18.9%, *p* < 0.04
**Paydas 2005 (^)** [70]	Effects of C0 vs. C2 Cyc monitoring in KTx	36	A: C0 (N = 25) B: C2 (N = 12)	Cl = A: 72.31 ± 23.1, B: 78.73 ± 22.42, *p* = 0.621	UA = 1 month: A: 7.94 ± 2.02, B: 6.14 ± 0.66, *p* = 0.008 6 months: A: 8 ± 2.2, B: 6.26 ± 0.93, *p* = 0.007 12 months: A: 7.88 ± 1.86, B: 6.1 ± 1.14, *p* = 0.005 24 months: A: 8.01 ± 1.73, B: 6.31 ± 0.99, *p* = 0.004 36 months: A: 8.66 ± 1.99, B: 6.82 ± 2.31, *p* = 0.065
**Pons 2009** [73]	Safety of Cyc withdrawal in LTx	10–132	A: Tolerant IS withdrawal (N = 8), B: Non-Tolerant IS withdrawal (N = 12)	35% with Cr > 1.3	UA = in 8 patients without rejection: 7.2 ± 1.8 -> 5.1 ± 1.1, *p* < 0.0001 in 12 patients with rejection:7.4 ± 1.6 -> 6.9 ± 1.5, *p* = 0.108
**Claes 2012** [76]	Metabolic parameters after KTx	12	A: Standard dose Cyc + MMF (N = 390), B: Low dose Cyc + MMF + CS (N = 399), C: Low dose Tac + MMF + CS (N = 401), D: Low dose Sir + MMF + CS (N = 399)	Not reported	UA = A: 7.2, D: 6, *p* < 0.05 B: 6.7, D: 6, *p* < 0.0001 C: 6.6, D: 6, *p* < 0.0001
**Einollahi 2013** [77]	Prevalence and risk factors of HU in KTx	36	HU group (N = 1340) No HU group (N = 2877)	Cr = 1.6 ± 0.9	HU = 31.8% (25%♂ and in 34%♀, *p* < 0.001) Cyc at C0: OR 1.0, 95%CI 1.002–1.006, *p* = 0.001 Cyc at C2: OR 0.99, 95%CI 0.998–1.001, *p* = 0.3
**Atbee 2022** [81]	Relationship between Cyc levels and toxicity	18	A: Cyc levels: C0 100 to 200 ng/mL (N = 34) B: Cyc levels: C0 > 200 ng/mL (N = 16)	Not reported	HU: A: 6%, B: 28%, *p* = 0.0001

UA = uric acid; HU = hyperuricemia; N = number of cases; KTx = kidney transplant; LTx = liver transplant; HTx = heart transplant; Cyc = cyclosporin; Aza = azathioprine; MMF = mycophenolate mofetil; CS = corticosteroids; Anti-Ly = antilymphocyte globulin; Cr = creatinine, reported in mg/dL; Cl = creatinine clearance, reported in mL/min; ♂ = male; ♀ = female; C0 = fasting cyclosporin level measured 12 h after the last dose; C2 = cyclosporin level measured 2 h after the morning dose. (^) Study evaluating both paired and unpaired groups.

**Table 3:** Descriptive items and results of studies that compared the effects of cyclosporin and tacrolimus on UA in patients after solid organ transplantation.

Study ID	Aim and Population	Follow-Up	Study Groups	Baseline Cr or Cl	Results
**Within-patient comparisons**					
**Van Thiel 1990** [51]	Gastrointestinal and metabolic effect of Cyc and Tac after LTx	<1	Before -> after LTx A: No IS -> Cyc (N = 20) B: No IS -> Tac (N = 20)	Not declared	UA = A: 4.2 ± 0.5 -> 6.2 ± 0.9, *p* = 0.063 B: 4.5 ± 0.5 -> 7.8 ± 1.0, *p* = 0.007 A: before LTx 4.2 ± 0.5 vs. B: Before LTx 4.5 ± 0.5, *p* = NS A: after LTx 6.2 ± 0.9 vs. B: after LTx 7.8 ± 1.0, *p* = NS
**Jordan 1994** [53]	Impact of the switch from Cyc to Tac in resistant KTx rejection	0.5–36	A: Cyc -> B:Tac (N = 77)	Cr = 2.35 ± 0.97 mg/dL	UA = A: 7.3 ± 2.3, B: 7.1 ± 1.5, *p* = 0.53
**Urbizu 2003** [63]	Efficacy and safety of conversion from Cyc to Tac in KTx	6–12	A: Cyc-> B:Tac (N = 55)	Cr stable. Values not reported	UA decreased in B, *p* = 0.005. Values not reported
**Hohage 2005** [68]	Effect of a conversion to Tac from Cyc in severely damaged KTx	36	A: Cyc (N = 30) B: Tac (N = 30)	Cr = A: 2.9, B: 2.2	UA = A:7.0 ± 0.1, B:6.4 ± 0.1, *p* < 0.05
**Kanbay 2005 (^)** [69]	Effects of Cyc and Tac on UA in KTx	24	Cyc -> Tac (N = 35)	Not reported	UA = 8.6 ± 2.8 -> 8.1 ± 1.9, *p* > 0.05
**White 2004 (^)** [82]	The impact of the switch from Cyc to Tac in Stable HTx with LDL > 2.5 mmol/L	6 months	Cyc -> Tac (N = 65)	Cl = A: 65.9 ± 23.8 B: 61.3 ± 9.9, *p* = NS	Δ = UA _at follow-up_ − UA _baseline_ 1 month: Δ 0.12 ± 0.94 -> 3 month: B: Δ −0.35 ± 1.06-> 6 months: B: Δ −0.5 ± 1.2, not reported
**Seymen 2009** [75]	Effect of conversion from Cyc to Tac on Hyperlipidemia in KTx	12	A: Cyc -> B: Tac (N = 15)	Cr= A: 1.47 ± 0.38, B: 1.5 ± 0.45, *p* = NS	UA mg/dL: A: 7.61 ± 1.84 B: 6.69 ± 1.35, *p* = 0.01
**Between-group comparisons**					
**Boots 2001** [57]	The impact of Tac vs. Cyc on graft function and on CV risk in KTx	12	A: Cyc + Pred (N = 74) B: Tac + Pred (N = 54)	Average Cl 47 mL/min A vs. B, *p* = NS	Fractional UA clearance no significant difference *p* > 0.25
**Neal 2001** [58]	Prevalence of HU in LTx	presumably 48	A: Cyc (N = 88) B: Tac (N = 43)	In HU: Cr= A: 1.9 ± 0.2, B: 1.54 ± 0.05, *p* = 0.039	HU: A: 51%, B: 42%, *p* = NS
**Balal 2004** [64]	Comparison of the effects of Tac and Cyc in KTx	24	A: Cyc + Pred + MMF or Aza (N = 11) B: Tac + Pred + MMF or Aza (N = 19)	Cl = A: 78.7 ± 22.4, B: 68.6 ± 27.1, *p* = NS	UA = 1 month: A: 6.1 ± 0.6 B: 5.8 ± 1.6, *p* = NS 6 months: A: 6.2 ± 0.9, B: 6.1 ± 1.3, *p* = NS 12 months: A: 6.1 ± 1.1, B: 6.5 ± 1.4, *p* = NS 24 months: A: 6.3 ± 0.9, B: 6.5 ± 0.8, *p* = NS
**Shibolet 2004** [65]	Incidence of HU and gout in HTx and LTx	36	A: LTx (N = 75) B: HTx (N = 47)	Cl = A: 61.9 ± 3.9, B: 83.9 ± 4	HU: A: 85.7%, B: 100%, *p* = 0.007 Average UA = A: 6.8 ± 0.14, B: 7.6 ± 0.23 *p* = 0.003
**Kanbay 2005 (^)** [69]	Effects of Cyc and Tac on UA in KTx	24	A: Cyc (N = 73) B: Tac (N = 47)	Not reported	UA = A: 1 month 6.3± 1.6 -> 2 years 7.9 ± 1.98, *p* < 0.001 B:1 month 6.5 ± 1.8 -> 2 years 8.0 ± 1.8, *p* < 0.001
**Sessa 2009** [74]	Effect of immunosuppressive regimens on cardiovascular risk factors in KTx	Not applicable	A: Tac + MMF + CS (N = 16) B: Tac + MMF (N = 12) C: Tac + CS (N = 14) D: Cyc + MMF + CS (N = 19) E: Cyc + MMF (N = 12) F: Cyc + CS (N = 12) G: Cyc + Eve + CS (N = 10) H: Sir + MMF + CS (N = 8)	Not declared	HU = D: 21%, A: 0%, *p* = NS E: 33%, H: 8%, *p* = NS F: 41%, C: 7%, *p* = NS G: 10%, F: 41%, *p* = NS G: 10%, D: 21%, *p* = NS G:10%, H: 62%, *p* < 0.05 D:21%, H: 62%, *p* = NS
**Claes 2012** [76]	Assessment of metabolic syndrome in the first year after KTx	12	A: Standard-dose Cyc (N = 390), B: Low-dose Cyc + MMF (N = 399), C: Low dose Tac + MMF (N = 401),	Not reported	Average UA = A: 7.2, B: 6.7, *p* = NS A: 7.2, C: 6.6, *p* < 0.05
**Malheiro 2012** [43]	Prevalence of HU and associated risk factors in KTx	91 (27–170)	A: No HU B: HU in Cyc + CS and/or MMF (N = 147, 48.7%) in Tac + CS and/or MMF (N = 149, 49.3%)	Cl= No HU: 57.2 ± 18.8 HU: 44.7 ± 15.4	In Cyc: A: 39.4%, B: 61.4%, *p* < 0.001 In Tac: A: 58.9%, B: 36.2%, *p* < 0.001 Cyc use vs. Tac OR 2.44 (95% CI 1.05–5.7)
**Harada 2016** [79]	The effects of high-dose MZ with a CNI in ABO incompatible KTx	24	A: Cyc (C0 levels < 200 ng/mL) (N = 22) B: Tac (N = 15)	Cr = A: 1.38 ± 0.41 B: 1.26 ± 0.35 *p* = NS	UA: A: 5.5 ±1.3, B: 6.4 ± 1.2, *p* = NS
**Azizzadeh 2020** [80]	Effect of early pre-emptive conversion from Cyc to Tac in KTx	12	A: Cyc + MMF + CS (N = 125) B: Conversion to Tac + MMF + CS (N = 41)	Cl = A: 66.15 ± 26.22, B: 67.82 ± 20.93, *p* = NS	Higher UA in B, *p* = 0.016
**White 2004 (^)** [82]	The impact of the switch from Cyc to Tac in Stable HTx with LDL > 2.5 mmol/L	6	A: Cyc -> Cyc (N = 64) B: Cyc -> Tac (N = 65)	Cl = A: 65.9 ± 23.8 B: 61.3 ± 9.9, *p* = NS	UA = Baseline: A: 7.1 ± 1.4, B: 7.2 ± 0.4, *p* = 0.688 Δ = UA _at follow-up_ − UA _baseline_ 1 month: A: Δ 0.1 ± 0.9, B: Δ 0.12 ± 0.94, *p* = 0.547 3 month: A: Δ 0.2 ± 0.9, B: Δ −0.35 ± 1.06 *p* = 0.006 6 months: A: Δ 0.01 ± 1, B: Δ −0.5 ± 1.2 *p* = 0.017

UA = uric acid; HU = hyperuricemia; N = number; KTx = kidney transplant; HTx = heart transplant; LTx = liver transplant; Cyc = cyclosporin; Aza = azathioprine; Pred = prednisone; CS = corticosteroid; MMF = mycophenolate mofetil; Cr = creatinine; Cl = creatinine clearance; LDL = low-density lipoprotein cholesterol. (^) Study evaluating both paired and unpaired groups.

**Table 4:** Summary of quality assessment for the type of studies.

Study Type	Number of Studies	Average Quality Assessment	Sample Size Range	UA Relevance Score IQR
Cross-sectional	5	Moderate	103–302	56–60
Retrospective Cohort	13	Low-Moderate	16–4217	33–68
Prospective Cohort	9	Moderate	15–50	37–58
RCT	9	High	21–1645	57–73

RCT = randomized controlled study, IQR = interquartile range.

**Table 5:** The quality and relevance assessment for each study in the analysis.

ID Study	CASP Quality Assessment	Strengths	Limits	Uric Acid as a Primary Outcome	UA Relevance Score
**Najarian 1985** [47]	High quality	Consistent sample size	Reporting reflects standards of the time, no analysis of uricosuric drugs Not blinded	No	68
**West 1987** [48]	Low quality	Precursor study	Small control group, risk of bias	Yes	48
**Gores 1988** [49]	Moderate quality	RCT	UA levels were not reported; only the prevalence of HU was provided. No data on kidney function	Yes	62
**Lin 1989** [50]	Moderate quality	Early descriptive evidence	Unable to establish temporality or causality. Baseline kidney function differed between groups	Yes	67
**Van Thiel 1990** [51]	Low quality		Small sample size Use of a historical comparison group High risk of bias	No	37
**Burack 1992** [52]	Moderate quality	Clear objective	Retrospective nature limits control over bias; all patients received Cyc	Yes	64
**Jordan 1994** [53]	Low quality	Conversion strategy clearly described	No randomization; high risk of bias	No	47
**Islam 1995** [54]	Moderate quality	Long-term exposure assessment	Limited methodological details	No	41
**Hilbrands 1996** [55]	Moderate quality	Conversion strategy clearly described	Randomization is questionable given the unequal number of recruited patients (12 vs. 9) and the presence of possible confounding factors	No	55
**Hansen 1998** [56]	Moderate quality	Detailed renal function assessment	Differences between the Cyc and Aza groups were not reported; temporality and causality could not be established	No	60
**Boots 2001** [57]	Low quality	Detailed clinical assessment	Limited description of secondary outcome, especially for UA	No	37
**Neal 2001** [58]	Low quality	Considered uricosuric agents	Retrospective, risk of bias Not designed for the differences in CNI therapy	Yes	57
**Pons 2001** [73]	Moderate quality	Long-term follow-up	Possible confounding factors, such as kidney function changes	No	52
**Schlitt 2001** [59]	Moderate to high quality	Clear study design	Not blinded Limited sample size	No	57
**Seymen 2001** [75]	Low quality		Observational Discrepancy in the conclusion about UA and the results	No	41
**Abdelrahman 2002** [60]	Low quality	Long follow-up period	No clear definition of IS therapy, limited methodological details	Yes	40
**Morales 2002** [61]	High quality	Clear kidney outcome	Kidney function was different, no analysis of uricosuric drugs Not blinded	No	75
**Pascual 2003** [62]	High quality	Sample size evaluation	Not blinded UA not reported as secondary outcome	No	62
**Urbizu 2003** [63]	Low quality		High risk of bias. Methods and results poorly reported	No	33
**Balal 2004** [64]	Moderate quality	Adequate follow-up and outcome reporting	No randomization and possible confounding factors. Some inconsistencies in follow-up and results reporting	No	52
**Shibolet 2004** [65]	Low quality		Results inconclusive, only speculative	Yes	35
**Wong 2004** [66]	Moderate quality	Well-defined intervention and outcomes	Small sample size Short follow-up Not blinded	No	62
**Bumbea 2005** [67]	Moderate quality	Detailed clinical assessment Clear conversion protocol	Retrospective nature limits control over bias	No	51
**Hohage 2005** [68]	Moderate quality	Adequate follow-up	No randomization and possible confounding factors	No	57
**Kanbay 2005** [69]	Moderate quality	Clear outcome measurement	Basal kidney function not reported. Possible confounding factors	Yes	66
**Paydaş 2005** [70]	Moderate quality	Detailed description of UA levels during follow-up	Absence of randomization increases risk of bias	No	58
**White 2005** [71]	High quality	Randomization and multicenter design. Outcomes clearly defined	Not blinded	No	68
**Chen 2008** [72]	Low quality	Detailed clinical assessment	Case series; small sample size High risk of bias	No	16
**Sessa 2009** [74]	Low quality	Detailed immunosuppressive therapy	Small sample size Results poorly described	No	43
**Claes 2012** [76]	High quality	Robust randomization and a large sample size	Metabolic outcomes were secondary endpoints (sub-analysis), UA results poorly described, basal kidney function not reported Not blinded	No	55
**Malheiro 2012** [43]	Moderate quality	Focus on UA Long follow-up Good sample size	Retrospective analysis Lack of covariates (diuretics, Losartan) in the analysis	Yes	62
**Einollahi 2013** [77]	High quality	Large sample size	No comparison group. No details about blood examination schedule	Yes	78
**Faulhaber 2013** [78]	Moderate quality	Clear outcome Adequate follow-up	No control group, potential treatment-selection bias, two interventions—Cyc reduction and CS withdrawal	No	44
**Harada 2016** [79]	Moderate quality	Multicenter design strengthens external validity	Increased risk of selection bias and confounding. Small sample size	No	46
**Azizzadeh 2020** [80]	Low quality	Clear objective and defined outcomes	High risk of selection bias and confounding factors, limited evaluation of UA	No	41
**Atbee 2022** [81]	Low quality		No definition of IS therapy, limited methodological details	No	32

IS = immunosuppression, UA = uric acid, HU = hyperuricemia.

**Table 6:** Potential confounding factors affecting uric acid outcomes. Red: not considered; green: assessed and not associated with UA outcomes; orange: partially assessed or assessed but still potentially confounding.

ID Study	Kidney Function	Diuretics	Other IS	Drugs Affecting UA	Diet
Najarian JS et al. [47]					
West C et al. [48]					
Gores FP et al. [49]					
Lin HY et al. [50]					
Van Thiel DH et al. [51]					
Burack DA et al. [52]					
Jordan ML et al. [53]					
Islam IS et al. [54]					
Hilbrands LB et al. [55]					
Hansen JM et al. [56]					
Boots JMM et al. [57]					
Neal DA et al. [58]					
Schlitt HJ et al. [59]					
Abdelrahman M et al. [60]					
Morales JM et al. [61]					
Pascual M et al. [62]					
Urbizu JM et al. [63]					
Balal M et al. [64]					
Shibolet O et al. [65]					
Wong V et al. [66]					
Bumbea V et al. [67]					
Hohage H et al. [68]					
Kanbay M et al. [69]					
Paydas S et al. [70]					
White M et al. [71]					
Chen J et al. [72]					
Pons JA et al. [73]					
Sessa A et al. [74]					
Seymen P et al. [75]					
Claes K et al. [76]					
Malheiro J et al. [43]					
Einollahi B et al. [77]					
Faulhaber M et al. [78]					
Harada S et al. [79]					
Azizzadeh L et al. [80]					
Atbee MYNA et al. [81]					

**Table 7:** Analysis of heterogeneity.

Study ID	Population	Immunosuppression Therapy	Time from Transplantation (Months)	Follow-Up (Months)	Baseline Cr or Cl	Definition of Hyperuricemia
Najarian 1985 [47]	KTx	A: Cyc + CS vs. B: Aza + CS + Antily	Not reported	3–36	A: Cr = 1.9 ± 0.6 B: Cr = 1.5 ± 0.4 (mg/dL)	UA > 7.6 mg/dL in ♂ and >6.0 mg/dL in ♀
West 1987 [48]	KTx	A: Cyc + CS B: Aza + CS	Not reported	At least 12 months	Cr (mg/dL): A: 2.4 ± 1.07 B: 2.5 ± 1.25, *p* = NS	UA > 8.5 mg/dL in ♂ and >7.0 mg/dL in ♀
Gores 1988 [49]	KTx	A: Cyc + CS vs. B: Aza + CS + Antily	Not reported	4 years	Cr < 2 mg/dL	UA > 8 mg/dL Severe HU: UA > 14 mg/dL
Lin 1989 [50]	KTx	A: Cyc + CS vs. B: Aza + CS	A: 96 ± 1 B: 74 ± 5	Not applicable	Cr (mg/dL): A: 1.8 ± 0.1 B: 1.4 ± 0.1, *p* = 0.0001	UA > 7.9 mg/dL in ♂ and >6.7 mg/dL in ♀
Van Thiel 1990 [51]	LTx	Cyc vs. Tac Other immunosuppression drugs not declared	Not reported	<1 month	Not reported	
Burack 1992 [52]	HTx	Cyc, CS, Aza	1	5–29	34% of cases had Cr > 1.4 mg/dL	UA level of >7.5 mg/dL in ♀ and >8.5 mg/dL in ♂
Jordan 1994 [53]	KTx in rejection	A: Cyc + CS +/−Aza vs. B: Tac + CS +/−Aza	4.3 ± 6.3	From 2 weeks to 36 months, mean 14 months	Cr = 3.2 ± 1.6 mg/dL	
Islam 1995 [54]	KTx	A: Cyc + Aza + CS vs. B: Aza + CS	Not reported	4–15 years	Not declared	
Hilbrands 1996 [55]	KTx	A: Cyc + CS vs. B: Aza + CS	3	4 weeks	Cr (mg/dL) A: 1.3 ± 0.3 B: 1.4 ± 0.46	
Hansen 1998 [56]	KTx	A: Cyc + CS + Aza vs. B: Aza + CS	9–178	Not applicable	Cr < 2.04 mg/dL	
Boots 2001 [57]	KTx	A: Tac + CS vs. B: Cyc + CS	Not reported	12	Average Cl 47 mL/min	
Neal 2001 [58]	LTx	A: Cyc vs. B: Tac Aza (only in the first year after LTx) +/−CS	6	Presumed to be 48 months	In HU: Cr (mg/dL) A: 2 ± 0.2 B: 1.5 ± 0.05, *p* = 0.039	UA > 7.6 mg/dL in ♂ and >6 mg/dL in ♀
Schlitt 2001 [59]	LTx with CNI toxicity	A: CNI B: replacement by MMF	at least 6	6	Cr (mg/dL): A: 1.6 ± 0.2 B: 1.9 ± 0.6	
Seymen 2001 [75]	KTx	Not reported	78 ±43	12 months	Cr (mg/dL): A: 1.47 ± 0.38 B: 1.5 ± 0.45, *p* = 0.54	
Abdelrahman 2002 [60]	KTx	A: Cyc + CS + Aza B: Cyc + CS C: Cyc + Aza D: Cyc + MMF	at least 12	107	Cr (mg/dL): 1.3 ± 0.3	UA level of >6 mg/dL in ♀ and >8 mg/dL in ♂
Morales 2002 [61]	KTx	A: Cyc + Aza or MMF vs. B: Sir + Aza or MMF	Not reported	104	Two years from KTx: Cr (mg/dL) A: 1.58–1.7 B: 1.36–1.47, *p* < 0.05	According to laboratory reference values
Pascual 2003 [62]	KTx with Cr <2 mg/dL and proteinuria <1 g/day	A: Cyc + Pred + MMF vs. B: Cyc reduction + Pred + MMF	Mean 21–22	6	Cr (mg/dL): 1.35 ± 0.24	
Urbizu 2003 [63]	KTx	Cyc vs. Tac +MMF, CS use not declared	Time to conversion was 36.5 ± 34	6–12 months	Cr was reported as stable in the cohort, but values were not reported	
Balal 2004 [64]	KTx	A: Tac + CS + MMF or Aza vs. B: Cyc + CS + MMF or Aza	Not reported	24	Cl (mL/min) A: 78.7 ± 22.4 B: 68.6 ±27.1, *p* = NS	
Shibolet 2004 [65]	HTx and LTx	HTx: Cyc 95.6%, Tac 4.1%, Aza 66%, CS 100%, MMF 4.3% LTx: Cyc 35.1%, Tac 64.9%, Aza 5.2%, CS 59.2%, MMF 11.7%	Not reported	At least 36 months	Cr (mg/dL): HTx 1.72 vs. LTx 1.3, *p* < 0.001	
Wong 2004 [66]	KTx with stable renal allograft function	A: 50% reduction in Cyc dosage + MMF + Pred vs. B: standard Cyc dose + MMF + Pred	at least 12	6 months	Cl (mL/min): A: 72.7 ± 17.9 B: 66.9 ± 19.6	
Bumbea 2005 [67]	KTx with chronic allograft dysfunction	Switch from Tac or Cyc to Sir +CS +/−Aza +/−MMF	Median 54 (6–192)	24	Cl (mL/min): 49.4 ± 14.9	
Hohage 2005 [68]	KTx	A: Cyc vs. B: Tac + CS + MMF (not described in the results)	at least 36	72 months (36 months before and 36 months after Tx)	Cr (mg/dL): A: Cr 2.9 B: Cr 2.2	
Kanbay 2005 [69]	Stable KTx	A: Cyc B: Tac C: from Cyc to Tac Other IS treatment not reported	1, 6, 12, 18, 24	From 1 to 24 months	Cr (mg/dL): <1.5	
Paydas 2005 [70]	KTx	A: C0 monitoring vs. B: C2 monitoring +Aza or MMF + CS	1	36	Cl (mL/min): A: 72.31 ± 23.1 B: 78.73 ± 22.2	
White 2005 [71]	Stable HTx	A: A: Cyc; 47% received Aza, 27% MMF, and 52% CS vs. B: Tac: 51% received AZA, 28% MMF, 52% CS	at least 12	6 months	Cl (mL/min): A: 65.9 ± 23.8 B: 61.3 ± 9.9, *p* = 0.333	
Chen 2008 [72]	KTx with chronic allograft nephropathy	Tac or Cyc switch to Sir +MMF + CS	At least 6	12	Cr (mg/dL): 3.2	
Pons 2009 [73]	LTx	Cyc was gradually withdrawn and followed later by CS and/or Aza (over two months) In patients with rejection, reintroduction of IS	24	10–132	Cr (mg/dL): Without Rejection: 1.54 ± 0.3 With rejection: 1.31 ± 0.27	
Sessa 2009 [74]	KTx	A: Tac + MMF + CS (N = 16) B: Tac + MMF (N = 12) C: Tac + CS (N = 14) D: Cyc + MMF + CS (N = 19) E: Cyc + MMF (N = 12) F: Cyc + CS (N = 12) G: Cyc + Eve + CS (N = 10) H: Sir + MMF + CS (N = 8)	Mean A: 54 B:57 C:62 D: 82 E:32 F:128 G: 66 H: 118	Not applicable	Not reported	As abnormal value
Claes 2012 [76]	KTx	A: Cyc high dose, B: Cyc low dose + MMF C: Tac low dose + MMF D: Sir low dose + MMF	Not reported	12	Not reported	
Malheiro 2012 [43]	KTx	77.8% in CS 74.8% in MMF 48.7% in Cyc 49.3% in Tac	91 (27–170)	Not applicable	Cl (mL/min) No HU: 57.2 ± 18.8 HU: 44.7 ± 15.4	UA > 7 mg/dL in ♂ and >6.5 mg/dL in ♀
Einollahi 2013 [77]	KTx	A: Cyc + MMF vs. B: Aza + CS	Mean 60 ± 48	Around 36	Cr (mg/dL): 1.6 ± 0.9	UA ≥7.0 mg/dL in ♂ and ≥6 mg/dL in ♀ that persisted for at least two consecutive tests.
Faulhaber 2013 [78]	HTx	CS withdrawn, MMF introduction, Cyc dose reduction (target level 50–90 ng/mL)	36	24	Cr (mg/dL): < 3.5	
Harada 2016 [79]	KTx ABO incompatible	A: Cyc B: Tac, +High-dose Mizoribine, basiliximab, rituximab, CS	Not reported	2 years	Cr (mg/dL) at 6 months from KTx A: 1.38 ± 0.41 B:1.26 ± 0.35, *p* = NS	
Azizzadeh 2020 [80]	KTx	A: Cyc + MMF vs. B: Tac + MMF	Not reported	12	Cl (mL/min): A: 66.15 ± 26.22 B: 67.82 ± 20.93, *p* = 0.757	
Atbee 2022 [81]	KTx	Cyc, other IS agents not reported	3	18	Not reported	Not defined

Tx = transplant; KTx = kidney transplant; LTx = liver transplant; HTx = heart transplant; Cyc = cyclosporin; Tac = tacrolimus; Aza = azathioprine; MMF = mycophenolate mofetil; Sir = sirolimus; CS = corticosteroid; CNI = calcineurin inhibitors; Cr = creatinine; Cl = creatinine clearance.

**Table 8:** Summary of findings and certainty in solid organ recipients in CNI treatment.

Outcome	Evidence Base (Studies, Participants)	Summary of Findings	Certainty (GRADE)	Reasons for Rating
CNI vs. non-CNI				
**HU** **(prevalence/incidence)**	9 studies; 5158 participants	HU was common in patients with CNI therapy; across studies, prevalence ranged broadly (≈30–80%), and switching/withdrawal or use of non-CNI regimens was generally associated with lower hyperuricemia burden.	Very low	Risk of bias: predominantly observational/retrospective + confounding risk factors (kidney function and diuretics) incompletely addressed Inconsistency: clinical heterogeneity; varying definitions of UA Indirectness: variable follow-up and IS treatment Imprecision: several small samples Suspected selective outcome/reporting: UA often secondary outcome
**UA level (continuous)**	6 comparative studies; 2066 participants; 10 cohort studies (reduction or stopping CNI); 306 participants	Treatment with CNI was often linked to higher UA levels. Reducing, stopping, or switching to non-CNI regimens usually improved UA during follow-up. However, these results varied depending on the study design and clinical setting.	Very low	Risk of bias: confounding risk factors (kidney function and diuretics) not systematically adjusted Inconsistency: mixed directions/contexts Indirectness: variable baselines and transplant vintage Imprecision: limited power in multiple cohorts Potential selective reporting: UA not consistently a primary endpoint
Cyclosporin vs. tacrolimus				
**HU** **(prevalence/incidence)**	4 comparative studies; 661 participants	Overall, there was no clear difference in HU prevalence. Some studies found higher rates with Cyc than with Tac, while others found no difference.	Very low	Risk of bias: nonrandomized comparisons; residual confounding) Inconsistency: contradictory findings Indirectness: mixed organ types/therapies; variable definitions Imprecision: small sample sizes in several studies Selective reporting likely: UA frequently secondary
**UA level (continuous)**	12 comparative studies; 2052 participants	Several studies found that cyclosporin was more often associated with higher UA levels than tacrolimus, whereas a similar number found no significant difference. Conversion from Cyc to Tac generally showed stable or reduced UA, with exceptions.	Very low	Risk of bias: confounding and baseline imbalance Inconsistency: heterogeneous and discordant direction Indirectness: variable follow-up and co-therapies Imprecision: few events and limited precision Suspected reporting bias: UA not uniformly/fully reported

CNI = calcineurin inhibitor, Cyc = cyclosporin, Tac = tacrolimus, IS = immunosuppression, UA = uric acid, HU = hyperuricemia.

## Data Availability

No new data were created or analyzed in this study.

## Abbreviations

The following abbreviations are used in this manuscript:

UA	Uric acid
CKD	Chronic kidney disease
CNI	Calcineurin inhibitor
PICO	Population, intervention, comparison, outcome
CASP	Critical Appraisal Skills Programme
RCT	Randomized controlled trial

## Appendix A

**Table A1:** Prisma 2020 Checklist.

Section and Topic	Item #	Checklist Item	Location Where Item Is Reported
**TITLE**			
Title	1	Identify the report as a systematic review.	1st page
**ABSTRACT**			
Abstract	2	See the PRISMA 2020 for Abstracts checklist.	1st page
**INTRODUCTION**			
Rationale	3	Describe the rationale for the review in the context of existing knowledge.	2nd and 3rd pages
Objectives	4	Provide an explicit statement of the objective(s) or question(s) the review addresses.	3rd page
**METHODS**			
Eligibility criteria	5	Specify the inclusion and exclusion criteria for the review and how studies were grouped for the syntheses.	3rd page
Information sources	6	Specify all databases, registers, websites, organisations, reference lists and other sources searched or consulted to identify studies. Specify the date when each source was last searched or consulted.	3rd page
Search strategy	7	Present the full search strategies for all databases, registers and websites, including any filters and limits used.	3rd page
Selection process	8	Specify the methods used to decide whether a study met the inclusion criteria of the review, including how many reviewers screened each record and each report retrieved, whether they worked independently, and if applicable, details of automation tools used in the process.	3rd page
Data collection process	9	Specify the methods used to collect data from reports, including how many reviewers collected data from each report, whether they worked independently, any processes for obtaining or confirming data from study investigators, and if applicable, details of automation tools used in the process.	4th page
Data items	10a	List and define all outcomes for which data were sought. Specify whether all results that were compatible with each outcome domain in each study were sought (e.g., for all measures, time points, analyses), and if not, the methods used to decide which results to collect.	4th page
	10b	List and define all other variables for which data were sought (e.g., participant and intervention characteristics, funding sources). Describe any assumptions made about any missing or unclear information.	4th page
Study risk of bias assessment	11	Specify the methods used to assess risk of bias in the included studies, including details of the tool(s) used, how many reviewers assessed each study and whether they worked independently, and if applicable, details of automation tools used in the process.	4th page
Effect measures	12	Specify for each outcome the effect measure(s) (e.g., risk ratio, mean difference) used in the synthesis or presentation of results.	4th page
Synthesis methods	13a	Describe the processes used to decide which studies were eligible for each synthesis (e.g., tabulating the study intervention characteristics and comparing against the planned groups for each synthesis (item #5)).	5th page
	13b	Describe any methods required to prepare the data for presentation or synthesis, such as handling of missing summary statistics, or data conversions.	5th page
	13c	Describe any methods used to tabulate or visually display results of individual studies and syntheses.	4th page
	13d	Describe any methods used to synthesize results and provide a rationale for the choice(s). If meta-analysis was performed, describe the model(s), method(s) to identify the presence and extent of statistical heterogeneity, and software package(s) used.	4th page
	13e	Describe any methods used to explore possible causes of heterogeneity among study results (e.g., subgroup analysis, meta-regression).	4th page
	13f	Describe any sensitivity analyses conducted to assess robustness of the synthesized results.	NA
Reporting bias assessment	14	Describe any methods used to assess risk of bias due to missing results in a synthesis (arising from reporting biases).	4th page
Certainty assessment	15	Describe any methods used to assess certainty (or confidence) in the body of evidence for an outcome.	5th page
**RESULTS**			
Study selection	16a	Describe the results of the search and selection process, from the number of records identified in the search to the number of studies included in the review, ideally using a flow diagram.	5th page
	16b	Cite studies that might appear to meet the inclusion criteria, but which were excluded, and explain why they were excluded.	Figure 1
Study characteristics	17	Cite each included study and present its characteristics.	Table 1
Risk of bias in studies	18	Present assessments of risk of bias for each included study.	Table 5
Results of individual studies	19	For all outcomes, present, for each study: (a) summary statistics for each group (where appropriate) and (b) an effect estimate and its precision (e.g., confidence/credible interval), ideally using structured tables or plots.	Table 2 and Table 3
Results of syntheses	20a	For each synthesis, briefly summarise the characteristics and risk of bias among contributing studies.	7th–10th pages 16th–19th pages
	20b	Present results of all statistical syntheses conducted. If meta-analysis was done, present for each the summary estimate and its precision (e.g., confidence/credible interval) and measures of statistical heterogeneity. If comparing groups, describe the direction of the effect.	NA
	20c	Present results of all investigations of possible causes of heterogeneity among study results.	Table 7
	20d	Present results of all sensitivity analyses conducted to assess the robustness of the synthesized results.	9th and 17th pages
Reporting biases	21	Present assessments of risk of bias due to missing results (arising from reporting biases) for each synthesis assessed.	Figure 1
Certainty of evidence	22	Present assessments of certainty (or confidence) in the body of evidence for each outcome assessed.	Table 8
**DISCUSSION**			
Discussion	23a	Provide a general interpretation of the results in the context of other evidence.	34th–36th pages
	23b	Discuss any limitations of the evidence included in the review.	37th page
	23c	Discuss any limitations of the review processes used.	37th page
	23d	Discuss implications of the results for practice, policy, and future research.	36th page
**OTHER INFORMATION**			
Registration and protocol	24a	Provide registration information for the review, including register name and registration number, or state that the review was not registered.	3rd page
	24b	Indicate where the review protocol can be accessed, or state that a protocol was not prepared.	3rd page
	24c	Describe and explain any amendments to information provided at registration or in the protocol.	3rd page
Support	25	Describe sources of financial or non-financial support for the review, and the role of the funders or sponsors in the review.	38th page
Competing interests	26	Declare any competing interests of review authors.	38th page
Availability of data, code and other materials	27	Report which of the following are publicly available and where they can be found: template data collection forms; data extracted from included studies; data used for all analyses; analytic code; any other materials used in the review.	3rd–5th page

From: Page MJ, McKenzie JE, Bossuyt PM, Boutron I, Hoffmann TC, Mulrow CD, et al. The PRISMA 2020 statement: an updated guideline for reporting systematic reviews. BMJ 2021;372:n71. doi: 10.1136/bmj.n71 [44]. This work is licensed under CC BY 4.0. To view a copy of this license, visit https://creativecommons.org/licenses/by/4.0/ (accessed on 15 December 2025).
